# Angiotensin II type 1/adenosine A_**2A**_ receptor oligomers: a novel target for tardive dyskinesia

**DOI:** 10.1038/s41598-017-02037-z

**Published:** 2017-05-12

**Authors:** Paulo A. de Oliveira, James A. R. Dalton, Marc López-Cano, Adrià Ricarte, Xavier Morató, Filipe C. Matheus, Andréia S. Cunha, Christa E. Müller, Reinaldo N. Takahashi, Víctor Fernández-Dueñas, Jesús Giraldo, Rui D. Prediger, Francisco Ciruela

**Affiliations:** 1 Departamento de Farmacologia, Universidade Federal de Santa Catarina, Trindade, 88049-900 Florianópolis, SC Brazil; 2 Institut de Neurociències and Unitat de Bioestadística, Universitat Autònoma de Barcelona, Network Biomedical Research Center on Mental Health (CIBERSAM), Bellaterra, Spain; 3 Unitat de Farmacologia, Departament de Patologia i Terapèutica Experimental, Facultat de Medicina, IDIBELL-Universitat de Barcelona, L’Hospitalet de Llobregat, Spain; 4 Institut de Neurociències, Universitat de Barcelona, Barcelona, Spain; 5 PharmaCenter Bonn, Pharmaceutical Institute, Pharmaceutical Chemistry I, University of Bonn, Bonn, Germany; 6 Programa de Pós-graduação em Neurociências, Centro de Ciências Biológicas, Universidade Federal de Santa Catarina, Trindade, 88049-900 Florianópolis, SC Brazil

## Abstract

Tardive dyskinesia (TD) is a serious motor side effect that may appear after long-term treatment with neuroleptics and mostly mediated by dopamine D_2_ receptors (D_2_Rs). Striatal D_2_R functioning may be finely regulated by either adenosine A_2A_ receptor (A_2A_R) or angiotensin receptor type 1 (AT_1_R) through putative receptor heteromers. Here, we examined whether A_2A_R and AT_1_R may oligomerize in the striatum to synergistically modulate dopaminergic transmission. First, by using bioluminescence resonance energy transfer, we demonstrated a physical AT_1_R-A_2A_R interaction in cultured cells. Interestingly, by protein-protein docking and molecular dynamics simulations, we described that a stable heterotetrameric interaction may exist between AT_1_R and A_2A_R bound to antagonists (i.e. losartan and istradefylline, respectively). Accordingly, we subsequently ascertained the existence of AT_1_R/A_2A_R heteromers in the striatum by proximity ligation *in situ* assay. Finally, we took advantage of a TD animal model, namely the reserpine-induced vacuous chewing movement (VCM), to evaluate a novel multimodal pharmacological TD treatment approach based on targeting the AT_1_R/A_2A_R complex. Thus, reserpinized mice were co-treated with sub-effective losartan and istradefylline doses, which prompted a synergistic reduction in VCM. Overall, our results demonstrated the existence of striatal AT_1_R/A_2A_R oligomers with potential usefulness for the therapeutic management of TD.

## Introduction

Angiotensin II (AII) is a peptidic hormone that causes vasoconstriction through activation of angiotensin receptor type 1 (AT_1_R). Indeed, it is a key component of the renin-angiotensin system (RAS), which regulates blood pressure^1^. Accordingly, blocking AT_1_Rs with selective antagonists (i.e. losartan) constitutes the first-line therapy to deal with hypertensive patients^2^. Interestingly, AII is also synthesized in the brain, where its levels are much higher than those observed in plasma^3^. In addition, AT_1_Rs are expressed both in neurons and glial cells^4^. Thus, the existence of an endogenous brain angiotensin system has been postulated, which may respond to AII synthesized in and/or transported into the brain (for review see ref. 5). The function of AII in the brain has still not been fully elucidated. However, a role in the control of stress reaction and cerebral circulation, and in the mechanisms leading to brain ischemia, neuronal injury and inflammation has been demonstrated^5^. In addition, AT_1_R blockade reduced brain inflammation responses^6^ and had beneficial effects in processes involving microglial activation and neuroinflammation (such as animal models of Alzheimer’s disease, brain ischemia and multiple sclerosis) (for review see ref. 7). Similarly, in animal models of parkinsonism induced by neurotoxins 6-hydroxydopamine (6-OHDA) and 1-methyl-4-phenyl-1,2,3,6-tetrahydropyridine (MPTP), an increase in AII levels, with concomitant AT_1_R overactivation, has been observed^8–10^. On the other hand, the presence of RAS components in the basal ganglia in general and in the nigrostriatal system in particular has also been reported. Altogether, it has been postulated that brain RAS may be involved in dopaminergic degeneration, especially when the dopaminergic system is impaired, thus contributing to the pathogenesis and progression of dopaminergic-related pathologies such as Parkinson’s disease (PD).

The concept that cell surface receptors may physically interact forming oligomers appeared early in the eighties, while characterizing G protein-coupled receptors (GPCRs) for neurotransmitters^11, 12^. Notably, striatal dopaminergic receptors in general, and the dopamine D_2_ receptor (D_2_R) in particular, constitute the archetypal GPCR capable of forming receptor-receptor complexes. Indeed, the potential impact of these oligomers in pathophysiological conditions involving dopaminergic dysfunction has been extensively studied. Interestingly, the D_2_R has been shown to oligomerize with several GPCRs^13^, including the adenosine A_2A_ receptor (A_2A_R)^14^. The D_2_R-A_2A_R heteromer is expressed in GABAergic striatopallidal neurons and a reciprocal negative allosteric receptor-receptor interaction is defined as its “biochemical fingerprint”^15^. Noteworthy, the D_2_R-A_2A_R heteromer has been defined as a potential pharmacological target for pathologies associated with dysfunctional dopaminergic signaling, such as PD and schizophrenia. Indeed, A_2A_R antagonists (i.e. istradefylline) are currently used for PD treatment in Japan^16^. On the other hand, the D_2_R has also been shown to oligomerize with the AT_1_R in the striatum^17^, thus the potential use of AT_1_R ligands to modulate dopaminergic signaling has been postulated. Interestingly, early studies also indicated interactions between the adenosinergic and the angiotensinergic systems, for instance the antinociceptive effect of AII was related to that produced by adenosine A_1_ receptor agonists^18^. In addition, an A_2A_R- and AT_1_R-mediated synergistic interaction in the peripheral RAS was described^19, 20^. Thus, while adenosine was able to reverse the stimulatory effect of AII on Na^+^-ATPase activity in the renal proximal tubules via A_2A_R activation^21^, A_2A_R blockers reduced AII-mediated ROS formation via Nox2 (NADPH complex enzyme) in endothelial cells^20^. Conversely, AII potentiated the adenosine-induced contraction of afferent arterioles^22^, while losartan-mediated AT_1_R blockade abolished the adenosine-mediated reflex sympatho-excitatory response in the brachial artery^23^. Altogether, the aforementioned evidence highlights the need for a better understanding of the adenosinergic system-RAS interaction. Furthermore, this interaction may be relevant not only in the periphery but also in the brain, where a functional interplay with the dopaminergic system may occur.

Here, we study the possible existence, both in cultured cells and in mouse striatum, of a physical AT_1_R-A_2A_R interaction, which may be a potential target for managing dopaminergic-related disorders (i.e. tardive dyskinesia, TD). Also, we seek to characterize the most likely heteromeric receptor arrangement through protein-protein docking and long-timescale molecular dynamics (MD) simulations. Finally, we propose a novel multimodal treatment for TD based on the use of AT_1_R and A_2A_R antagonists at sub-effective doses, and test it in a mouse TD model, namely the reserpine-induced vacuous chewing movement (VCM).

## Results

### AT_1_R and A_2A_R form heteromers in cultured cells

**Figure 1:**
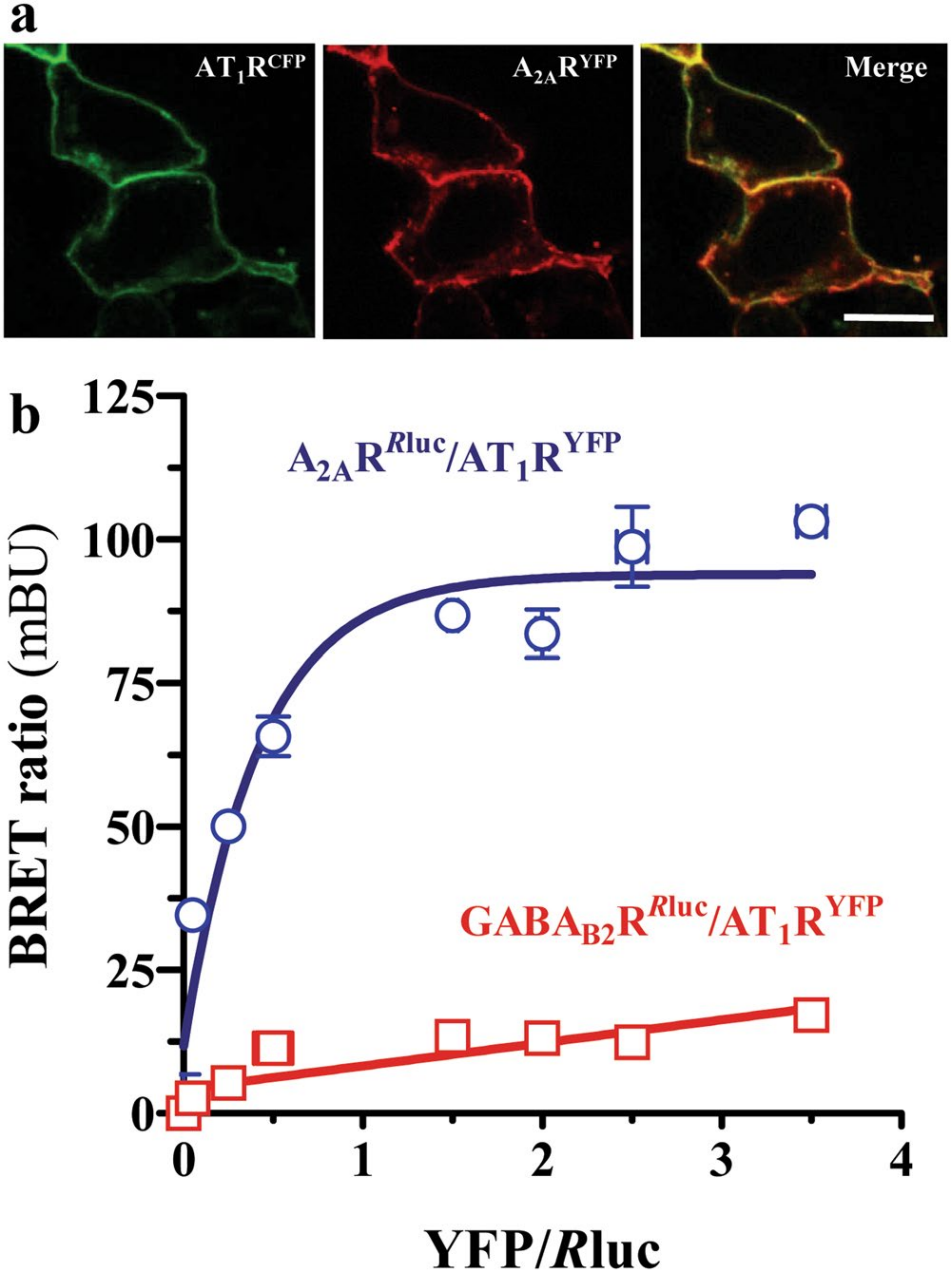
AT_1_R and A_2A_R physically interact in HEK-293T cells. (**a**) Co-distribution of AT_1_R and A_2A_R in HEK-293T cells. Transiently transfected HEK-293T cells with AT_1_R^YFP^ (red) and A_2A_R^CFP^ (green) were fixed and observed by confocal microscopy. Co-distribution (yellow) is shown in the merge image. *Scale bar*: 10 µm. (**b**). Direct interaction between AT_1_ and A_2A_ receptors. BRET saturation curves in HEK-293T cells expressing A_2A_R^*R*luc^ and AT_1_R^YFP^ (blue) or GABA_B2_R^*R*luc^ and AT_1_R^YFP^ (red). Plotted on the x-axis is the fluorescence value obtained from the YFP, normalized with the luminescence value of *R*luc-tagged vectors 10 min after benzyl-coelenterazine incubation. Results are expressed as mean ± SEM (n = 3, in triplicate).

Based on the existence of AT_1_R/D_2_R heteromers^17^, we aimed to elucidate whether AT_1_R is also able to oligomerize with the A_2A_R, a well-known D_2_R partner^24^. To this end, we first assessed the co-distribution of AT_1_R and A_2A_R in cultured cells through the fluorescence detection of CFP/YFP tagged receptors. Thus, by means of confocal microscopy analysis of HEK-293T cells transiently expressing AT_1_R^CFP^ and A_2A_R^YFP^, a high overlapping in the distribution of the former receptors was observed (Fig. 1a). Next, we examined the possible physical interaction of AT_1_R and A_2A_R in living cells by means of the BRET approach. Thus, cells were transiently transfected with receptor constructs carrying the appropriate fluorophore pairs (A_2A_R^*R*luc^ and AT_1_R^YFP^). A positive and saturable BRET signal was observed in cells co-transfected with a constant concentration of the A_2A_R^*R*luc^ and increasing concentrations of AT_1_R^YFP^ (Fig. 1b). Of note, as the control pair GABA_B2_R^*R*luc^ and AT_1_R^YFP^ led to a low and linear distribution, the specificity of the saturation (hyperbolic) assay for the A_2A_R^*R*luc^ and AT_1_R^YFP^ pair could be established (Fig. 1b). Overall, these results demonstrate that AT_1_R and A_2A_R form heteromers in living HEK-293T cells.

### Structure of AT_1_R/A_2A_R heteromer

Computational modeling, protein-protein docking, and MD simulations were used to probe the interaction between AT_1_R and A_2A_R, and determine their most likely heteromeric arrangement. Initially, AT_1_R and A_2A_R antagonists (losartan and istradefylline, respectively), were docked into their respective inactive-state receptor crystal structure using Autodock4.2^25^. The corresponding best docked AT_1_R-losartan and A_2A_R-istradefylline complexes were then embedded in lipid bilayer membranes and subjected to MD simulations of 250 ns and 500 ns, respectively, where both bound antagonists were observed to stabilize. In particular, in AT_1_R, ARG167, located on extracellular loop 2 (ECL2) above the orthosteric pocket, was observed to make H-bonds with losartan at both ends of the ligand (see SI Fig. 1) in a similar manner to that observed in the AT_1_R crystal structure containing bound olmesartan^26^. Likewise, in A_2A_R, ASN253 (ASN^6.55^ in Ballesteros-Weinstein numbering^27^) made an H-bond with istradefylline in a similar manner to other co-crystallized A_2A_R xanthine antagonists^28^ (Fig. S1).

As both AT_1_R and A_2A_R are thought to form functional homodimers at the cell surface^29–35^, we investigated the likely structure and behavior of these respective homodimers with bound antagonists, prior to investigating heteromeric interactions. In order to do this we utilized the A_2A_R homodimer crystal structure with co-crystallized antagonist^36^ as a structural guide for initializing AT_1_R-losartan and A_2A_R-istradefylline homodimer models. This dimeric crystal structure is observed to contain an interface between TM4 and TM5 helices of each monomer, with TM4 of one monomer interacting with TM5 of the other, and vice versa^36^. Initial AT_1_R and A_2A_R homodimer models were refined with protein-protein docking using the ROSIE webserver^37^, each consisting of two antagonist-bound receptors in the same MD-generated conformation (*see* above). Following protein-protein docking, the A_2A_R and AT_1_R homodimers were subjected to further MD simulations of 1.5 μs and 750 ns, respectively. During these simulations, both AT_1_R and A_2A_R homodimers were seen to form significant interactions via their TM4 and TM5 helices, respectively, with considerable contact between monomers, indicative of energetically stable dimers (Fig. S2). In addition, the respective bound antagonists remained stably bound in each participating monomer, with all receptor subunits maintaining an inactive state. From these results, it was inferred that the antagonist-bound homodimeric states of AT_1_R and A_2A_R are stable *in silico*, and likely form the minimum constituents that participate in cross-receptor heteromeric interaction.

**Figure 2:**
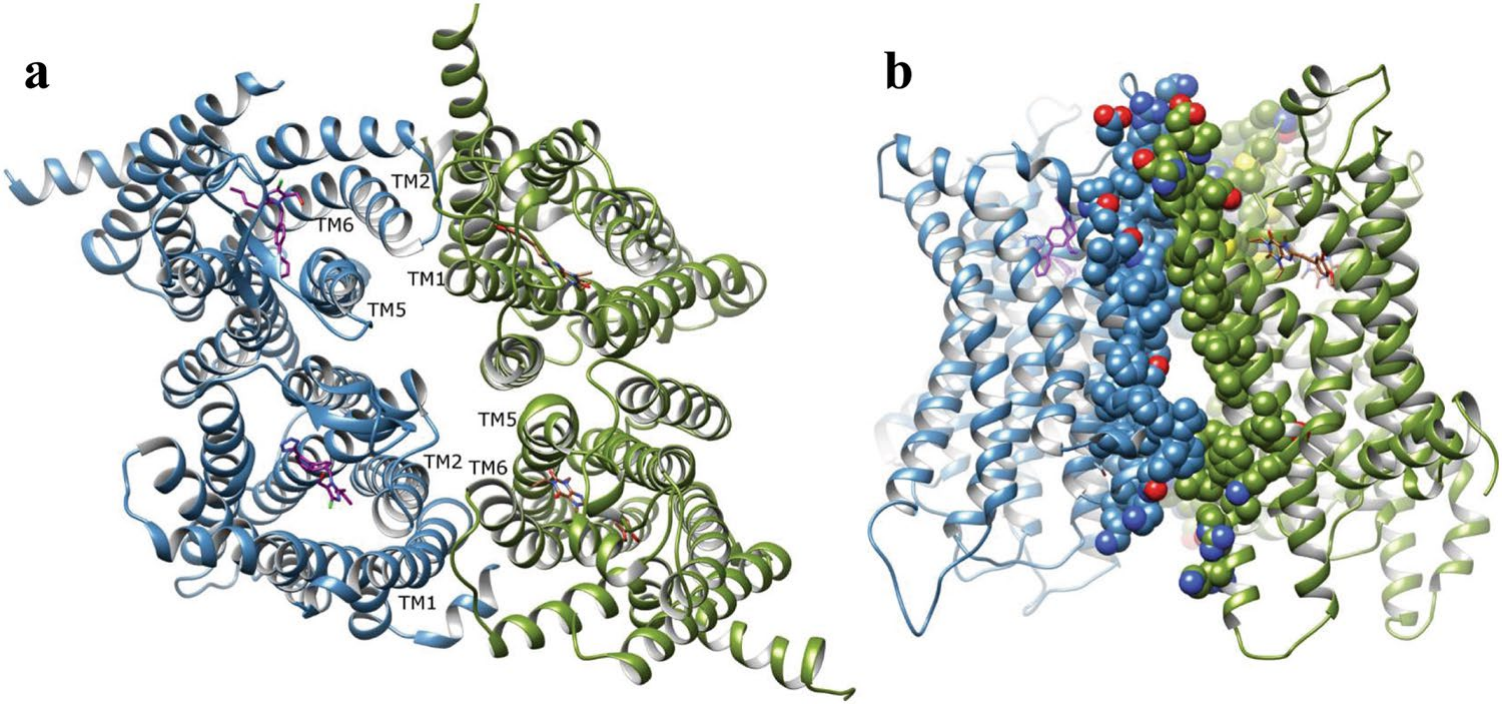
Conformational arrangement of AT_1_R/A_2A_R heterotetramer. Model generated by protein-protein docking and 2 μs MD simulation. (**a**) Top view of tetramer (AT_1_R in blue, A_2A_R in green, losartan in purple and istradefylline in brown). (**b**) Side view of interaction between A_2A_R and AT_1_R.

As other described heteromeric interactions involving A_2A_R fit a heterotetramer model^38, 39^, and as MD simulations of AT_1_R and A_2A_R homodimers suggest their respective stability, we investigated heterotetrameric interactions between the two receptor homodimers. As there is no crystal structure for GPCRs in tetrameric formation, we performed extensive protein-protein docking with ROSIE to identify the highest possible scoring interaction of AT_1_R and A_2A_R homodimers (*see* Methods). The “best” conformation identified a tetramer with cross-receptor interfaces involving TM5 and TM6 of one receptor with TM1 and TM2 of the other, and vice versa (Fig. 2). In order to assess the stability of the proposed interaction, the heterotetramer complex was subjected to an MD simulation in a membrane for 2 µs. Results show the receptors progressively stabilized (RMSD curve in Fig. S3) and enhanced their interaction, whilst maintaining the original tetrameric configuration (Fig. 2). Furthermore, the respective AT_1_R and A_2A_R homodimers remained stable and unperturbed within the tetramer, maintaining their respective inactive states. In conclusion, stable heterotetrameric interaction between AT_1_R and A_2A_R is plausible at a molecular level and compatible with bound antagonists, losartan and istradefylline.

### Functional consequences of the AT_1_R and A_2A_R oligomerization

**Figure 3:**
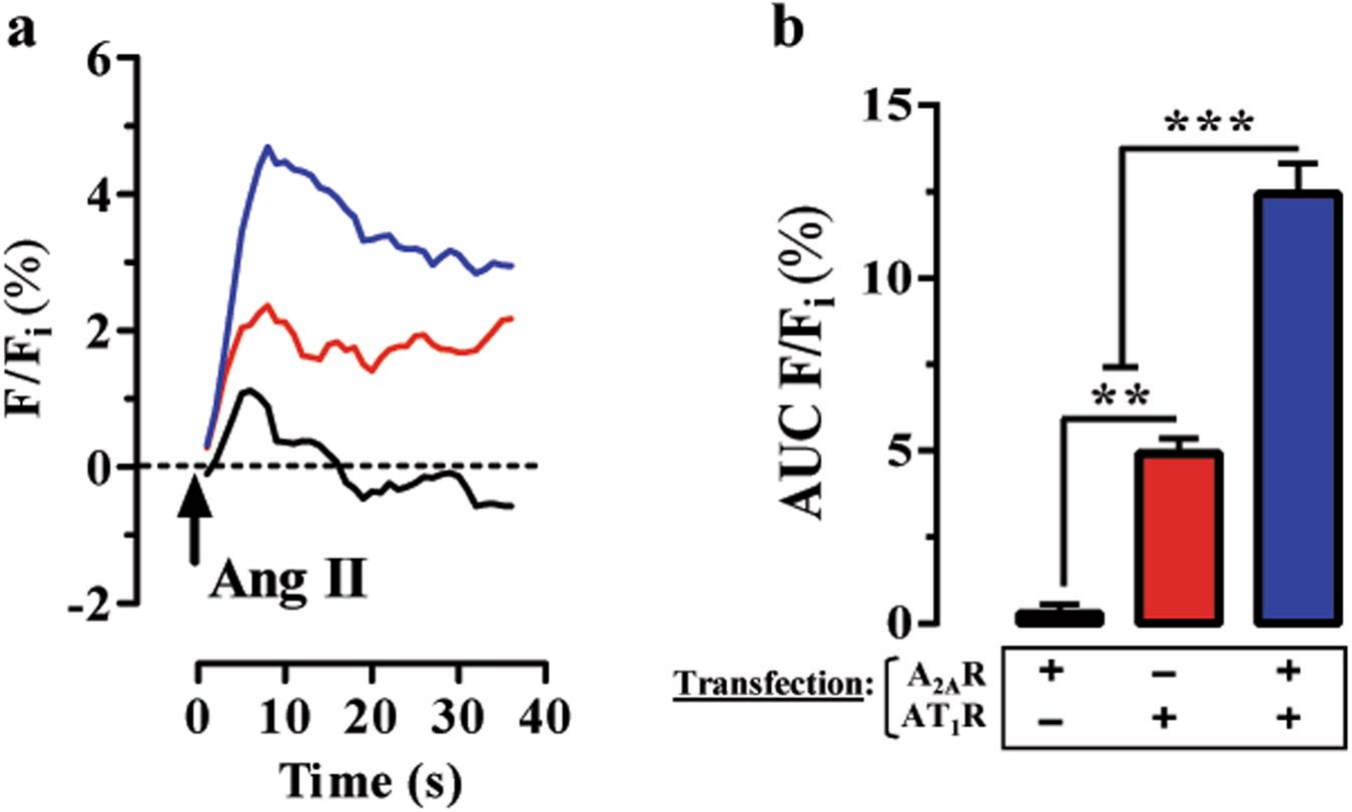
A_2A_R expression potentiates AT_1_R functioning. (**a**) Representative Angiotensin II-mediated intracellular Ca^2+^ accumulation determined by Fluo4 assay. HEK-293T cells were transiently transfected with A_2A_R (black trace), AT_1_R (red trace) and A_2A_R + AT_1_R (blue trace). Cells were loaded with Fluo4-NW dye and challenged with Angiotensin II (50nM). The [Ca^2+^]_i_ dynamics is shown as change in fluorescence of the Fluo4 signal (F) expressed as percentage of the maximal Ca^2+^ influx elicited by ionomycin (F_i_) in each experimental conditions. (**b**) Quantification of the AT_1_R-mediated [Ca^2+^]_i_ accumulation measured by Fluo4. The integrated area under the curve (AUC) of the normalized AT_1_R-mediated Fluo4 signal (F) is expressed as percentage of the corresponding ionomycin signal (Fi) for each transfection. The data are expressed as the mean ± SEM of three independent experiments performed in triplicate. The asterisk indicates statistically significant differences (**P < 0.01, ***P < 0.001; 1-way ANOVA with a Newman-Keuls *post-hoc* test).

The formation of AT_1_R-A_2A_R complexes in transfected cells suggests that there might exist a functional coupling between these two receptors. Thus, we assessed the impact of A_2A_R expression on AT_1_R-mediated intracellular Ca^2+^ mobilization from internal stores by means of Fluo4 determinations. Thus, in Fluo4 loaded cells expressing AT_1_R alone, the activation with angiotensin II increased intracellular Ca^2+^ (Fig. 3a, red trace), as expected. Interestingly, in cells co-expressing AT_1_R and A_2A_R, the angiotensin II-mediated intracellular Ca^2+^ mobilization was boosted (Fig. 3a, blue trace). Indeed, in cells expressing only A_2A_R, a residual and not significant effect of angiotensin II was observed, probably because of the endogenous expression of AT_1_R in HEK-293T cells (Fig. 3a, black trace). Quantification of the results (Fig. 3c) demonstrated a significant [F (2,6) = 8.40 (*P* < 0.05)] difference between the experimental groups assessed, thus a significant (*P* < 0.05) increase in the AT_1_R-mediated intracellular calcium accumulation in AT_1_R-A_2A_R cells was observed (Fig. 3c). These results suggest that a functional interplay between AT_1_R and A_2A_R might exist upon expression in heterologous cells.

### AT_1_R and A_2A_R heteromers are expressed in mouse striatum

**Figure 4:**
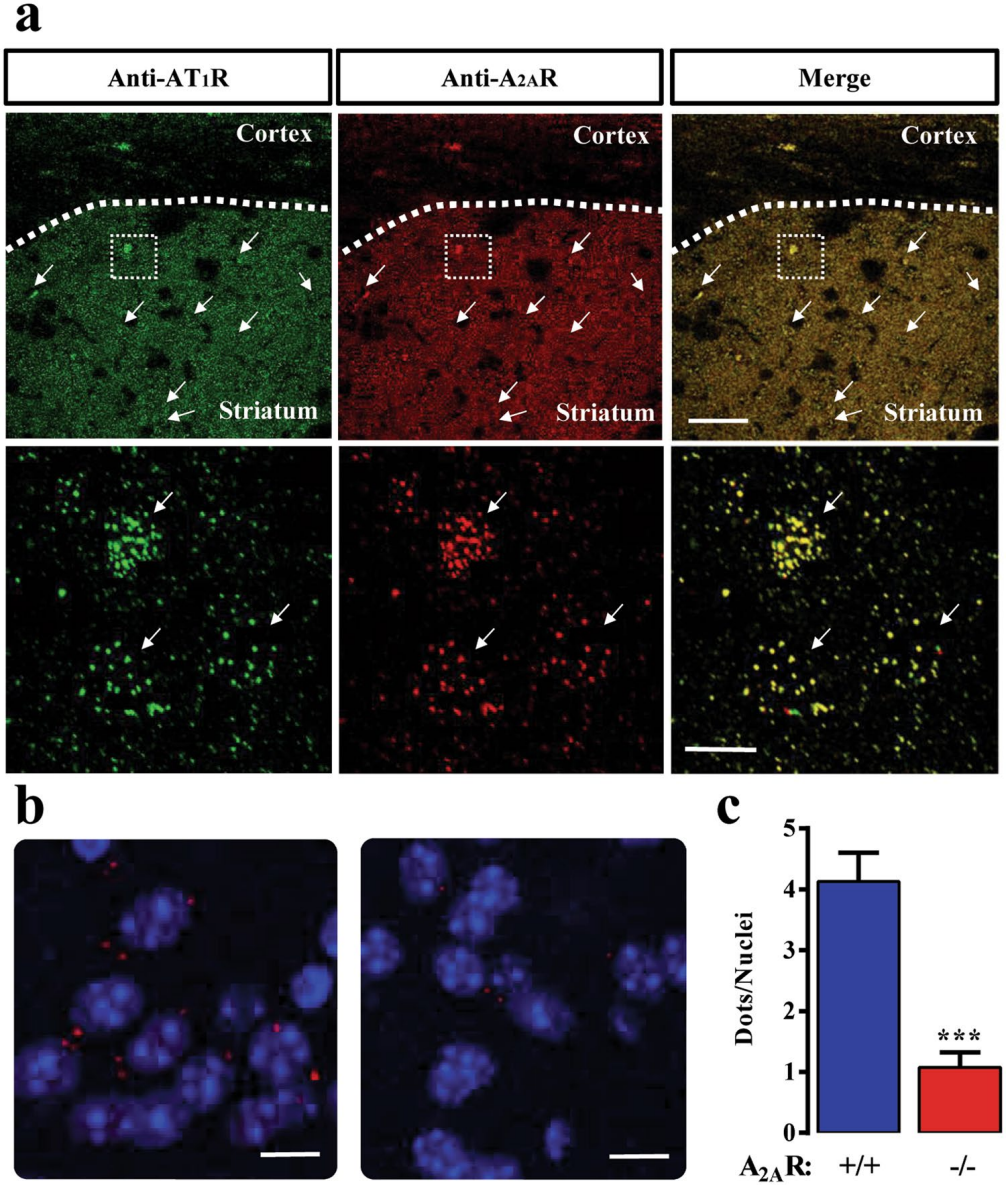
Detection of AT_1_R and A_2A_R proximity in mice striatal sections. (**a**) Immunohistochemistry detection of AT_1_R and A_2A_R in mice striatum. Representative confocal microscopy images of AT_1_R (red) and A_2A_R (green) immunoreactivities in the striatum are shown. Lower panels show a magnification of the square area shown in the upper panel. Arrows indicate potential location of medium spiny neurons (MSN) cell bodies. Superimposition of images revealed a high receptor co-distribution in yellow (merge). Scale bars: 350 μm (upper panels) and 10 μm (lower panels). (**b**) Photomicrographs of dual recognition of AT_1_R and A_2A_R with P-LISA. Representative images from wild-type (left) and A_2A_R-KO (right) mice striatum. (**c**) Quantification of P-LISA signals for AT_1_R and A_2A_R proximity confirmed the significant difference of P-LISA signal density between wild type and A_2A_R-KO mice (***P < 0.001). Values in the graph correspond to the mean ± SEM (dots/nuclei) of at list three animals and 5 images per animal. Scale bar: 10 μm.

Once demonstrated that AT_1_R and A_2A_R assemble into functionally interacting complexes in living cells, we aimed to determine the existence of AT_1_R/A_2A_R heteromers in native tissue, namely the striatum. To this end, we first conducted immunofluorescence experiments to assess the expression levels and distribution of both AT_1_R and A_2A_R in mouse striatum. Interestingly, both receptors showed a high degree of co-distribution throughout the striatal neuropil (Fig. 4a, upper panels) and eventually within the medium spiny neurons (MSN) cell bodies (Fig. 4a, lower panels). Importantly, the myelinated fiber bundles that penetrate the striatum were visible as dark (not stained) structures within the stained neuropil (Fig. 4a, upper panel). These results give rise to the possibility that these two receptors might be forming heteromers under native conditions. Subsequently, to confirm the existence of AT_1_R/A_2A_R heteromers in the striatum we implemented the P-LISA approach, a well described technique providing enough sensitivity to evaluate receptor’s close proximity within a named GPCR oligomer in native conditions^40^. Thus, by using proper antibody combinations, the AT_1_R/A_2A_R heteromer expression in mouse striatum was addressed by P-LISA assays. Indeed, red dots reflecting a positive P-LISA signal was observed in the striatum of wild-type mice (Fig. 4b), thus allowing the visualization of the AT_1_R/A_2A_R receptor-receptor interaction. Interestingly, in striatal slices from the A_2A_R-KO mice the P-LISA signal was negligible (Fig. 4b), thus reinforcing the specificity of our P-LISA assay. Indeed, when the P-LISA signal was quantified the wild-type animal showed 4 ± 0.5 dots/nuclei while the A_2A_R-KO displayed only 1 ± 0.2 dots/nuclei under the same experimental conditions. Thus, a marked and significant (*P* < 0.005) reduction in the P-LISA signal was observed in the A_2A_R-KO striatal slices. Taken together, data gathered from our P-LISA experiments strongly support the existence of AT_1_R/A_2A_R heteromers in the mouse striatum.

### Functional interplay between AT_1_R and A_2A_R in an animal model of TD

**Figure 5:**
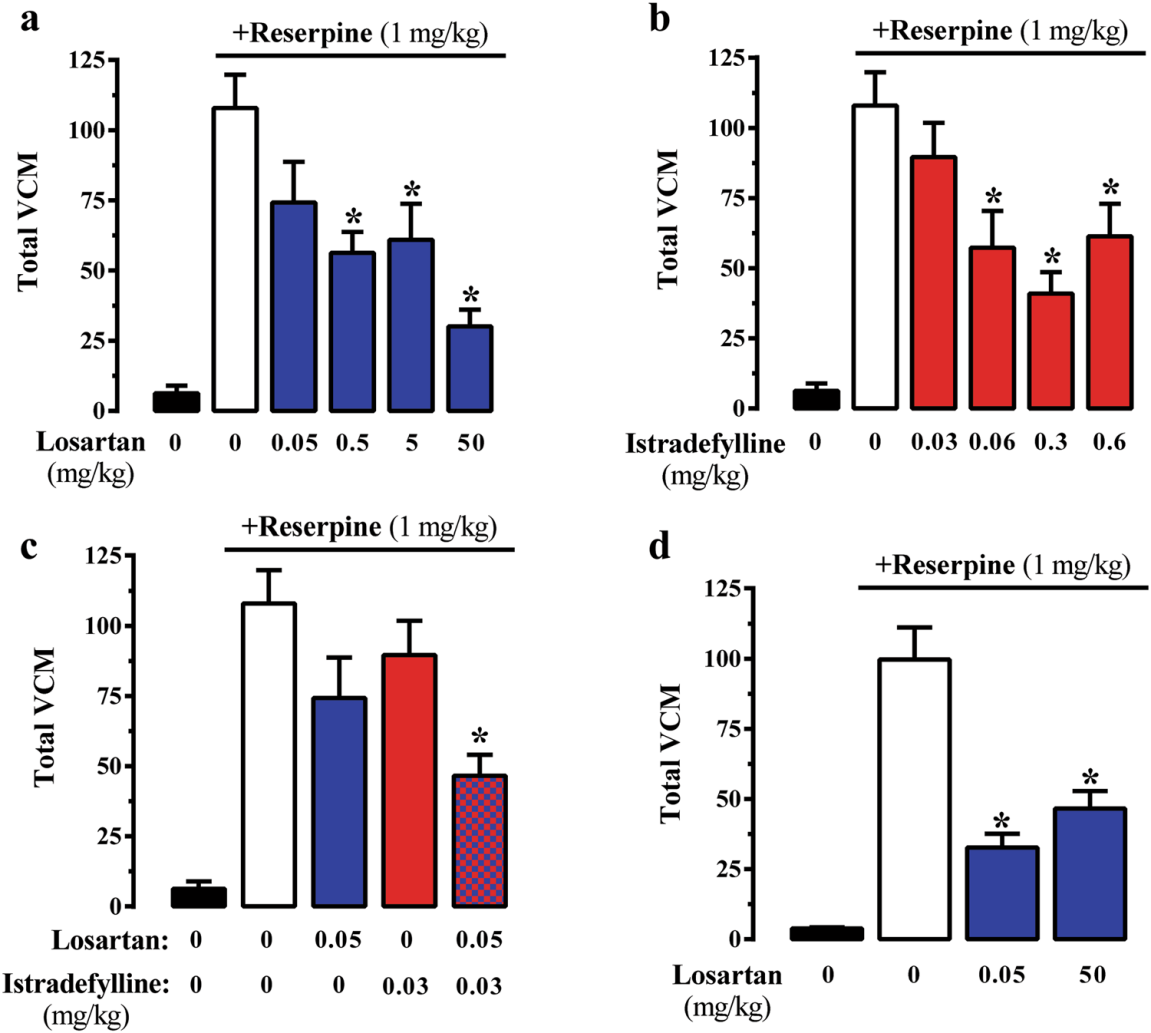
Effect of AT_1_R and A_2A_R blocking in the TD animal model. The effect of different doses of losartan (**a**) or istradefylline (**b**) on total vacuous chewing movements (VCM) in the reserpine-based animal model of TD in mice was monitored during 10 min. **(c)** Effects of sub-effective dose co-administration (i.p.) of losartan (0.05 mg/ml) and istradefylline (0.03 mg/ml) in the VCM of TD animal model. (**d**) Effect of losartan in the VCM of TD animal model performed in A_2A_R-KO mice. Results are represented as the mean ± SEM (n = 10 animals). *P < 0.05 compared to the vehicle group (one-way ANOVA, followed by Newman-Keuls test).

A_2A_R-containing oligomers, including A_2A_R/D_2_R^41^, are thought to be involved in the control of locomotor function both in normal and pathological conditions^42, 43^. However, although A_2A_R has been linked to neuroleptic-induced TD^44, 45^, its impact on this syndrome is still ambiguous^46^. Consequently, we sought to investigate whether the AT_1_R/A_2A_R heteromer might play a role in TD. We took advantage of the vacuous chewing movement (VCM) model of TD in mice. Interestingly, administration of the AT_1_R antagonist losartan dose-dependently reduced reserpine-induced VCM (Fig. 5a). Similarly, administration of the A_2A_R antagonist istradefylline dose-dependently reduced reserpine-induced VCM (Fig. 5b). Subsequently, we investigated whether co-treatment at sub-effective low doses of AT_1_R and A_2A_R antagonists would elicit a significant reduction of VCM in our reserpine-induced TD animal model. Therefore, for combination treatment, 0.05 mg/kg of losartan and 0.03 mg/kg of istradefylline were selected as they were not effective in reducing VCM. Noteworthy, the combined treatment produced a significant (*P* < 0.05) reduction in VCM (Fig. 5c), thus demonstrating a synergistic interaction between both drugs. Overall, these results suggest that co-treatment with AT_1_R and A_2A_R antagonists at sub-effective low doses is a useful therapeutic approach for TD management.

Finally, in an attempt to ascertain the role of AT_1_R/A_2A_R oligomers in the synergistic effect observed upon receptor antagonist co-treatment, we assessed the efficacy of the VCM sub-effective losartan dose in mice lacking the A_2A_R (i.e. A_2A_R-KO mice). Interestingly, the low dose of losartan (0.05 mg/kg) was able to significantly (*P* < 0.05) reduce the number of VCM in the A_2A_R-KO mice (Fig. 5d). Hence, in the absence of A_2A_R the efficacy of losartan was higher, thus indicating that AT_1_R/A_2A_R heteromers are crucial for finely modulating TD. Collectively, these results suggest that AT_1_R and A_2A_R functionally interact *in vivo* and that this functional interplay may be provided by the existence of AT_1_R/A_2A_R oligomers.

## Discussion

TD is a serious motor side effect associated to long-term treatment with neuroleptics^47^. Notably, D_2_R occupancy and its transience to occupation have been identified as a potential mechanistic substrate to develop antipsychotic-induced TD^48^. Indeed, D_2_R-mediated control of motor function has been related to the ability of this receptor to oligomerize with other GPCRs in general^49^ and with the A_2A_R in particular^42, 43, 50^. Also, in the brain, dopaminergic neurotransmission can be modulated by AII through AT_1_R. Thus, AT_1_R blocking precludes AII-mediated dopamine release^51, 52^. Furthermore, a functional interaction between angiotensin and dopamine receptors in the striatum and *substantia nigra* ^53, 54^, together with the formation of D_2_R and AT_1_R heteromers in the striatum has been described^17^. Based on these data, we decided to explore a possible direct interaction between AT_1_R and A_2A_R, and revealed for the first time the existence of AT_1_R/A_2A_R oligomers in the striatum and its implications in TD.

Our experimental data shows that AT_1_R and A_2A_R form heteromers both in co-transfected cells and in mouse striatum. This feature is especially strengthened by our *in-silico* analysis, which has predicted a heterotetrameric receptor arrangement that was stable during 2 μs of MD simulation. The “best” receptor-receptor interface identified for the AT_1_R/A_2A_R heterotetramer involves TM5 and TM6 of one receptor with TM1 and TM2 of the other, and vice versa, while in the respective homodimers the TM4 of one monomer interactac with TM5 of the other, and vice versa. Interestingly, the D_2_R/A_2A_R heterodimeric interface has been postulated to be formed by the TM4 and TM5 of D_2_R interacting with TM4 and TM5 of the A_2A_R^49, 55^. Therefore, when considering a putative AT_1_R/D_2_R/A_2A_R oligomer new in-silico analysis will be needed to accurately determine TM-TM contacts and receptor rearrangement defining AT_1_R/D_2_R/A_2A_R oligomer stoichiometry. Overall, this information will be extremely valuable when assessing potential multimodal TD pharmacotherapeutic interventions based on drugs targeting these receptors.

The AT_1_R/A_2A_R oligomerization was shown to elicit functional consequences, since co-expression with A_2A_R boosted AT_1_R signaling. This AT_1_R gain of function may most likely result from an A_2A_R-mediated AT_1_R increased cell surface targeting, as was previously reported^56^. Alternatively, an A_2A_R-mediated direct trans-activation of AT_1_R could not be excluded, as has been described for other A_2A_R-containing oligomers^41^. Thus, further work is needed to elucidate the precise molecular mechanism behind this AT_1_R/A_2A_R oligomer-dependent AT_1_R gain of function. Nevertheless, our main purpose consisted of ascertaining the *in vivo* implications of the AT_1_R/A_2A_R oligomer formation, which is the cornerstone when describing a new GPCR oligomer^57^. Indeed, our P-LISA data strongly supported the existence of AT_1_R/A_2A_R heteromers in the mouse striatum, thus warranting the need to assess the impact of this oligomer in behaving animals. Accordingly, we demonstrated an unprecedented synergism of AT_1_R and A_2A_R antagonists on the control of involuntary mandibular movements induced by reserpine in an animal model of TD. Thus, co-treatment with AT_1_R and A_2A_R antagonists at sub-effective low doses robustly (>60%) reduced reserpine-mediated VCM. Certainly, this makes this multimodal pharmacological approach an attractive solution for TD management.

The striatum is considered a pivotal brain region, since it receives projections from other basal ganglia areas and from many other brain regions involved in motor and non-motor functions, such as the motor cortex, the prefrontal cortex and the hippocampus^58, 59^. Indeed, both the renin-angiotensin and the adenosinergic systems play an important role in controlling the striatal function. Thus, the ability of AT_1_R and A_2A_R to heteromerize in the striatum might constitute a way of fine-tuning multiple receptor-signaling pathways harmonizing dopaminergic neurotransmission. Therefore, the AT_1_R/A_2A_R oligomer could be envisaged as a potential drug target for striatum-related adverse motor dysfunctions associated to therapy, including TD and L-DOPA induced dyskinesia (LID). Indeed, A_2A_R antagonists have been postulated and licensed as antiparkinsonian drugs^60^ and eventually studied in the management of LID^61^. Furthermore, A_2A_R has been linked to neuroleptic-induced TD^44, 45^, although with some debate^46^. Similarly, preclinical studies have demonstrated that blockade of AT_1_R reduces LID^62^. It is assumed that these A_2A_R- and AT_1_R-mediated anti-LID effects are related to their ability to heteromerize with D_2_R^17, 24^ and thus controlling dopaminergic neurotransmission. However, it could be speculated that AT_1_R and A_2A_R might control D_2_R function through functional AT_1_R/D_2_R/A_2A_R-containing complexes in GABAergic striatopallidal neurons. A number of facts support this last statement: i) the high and selective co-expression of AT_1_R, D_2_R and A_2A_R in these particular cells; ii) the demonstration of A_2A_R/D_2_R, AT_1_R/D_2_R and AT_1_R/A_2A_R heteromers; and iii) the existence of strong multiple interactions between the three receptors. In conclusion, the demonstration of their simultaneous physical interaction may constitute a novel and very attractive target for developing new drugs in the management of pathologies in which these receptors play a key role, such as TD.

## Methods

### Reagents

The primary antibodies used were: rabbit anti-AT_1_R polyclonal antibody (Abcam, Cambridge, UK), and mouse anti-A_2A_R monoclonal antibody (Millipore, Billerica, MA, USA). The secondary antibodies were: horseradish peroxidase (HRP)-conjugated goat anti-rabbit IgG (Pierce Biotechnology, Rockford, IL, USA) and Cy3-conjugated donkey anti-mouse IgG antibody (Jackson ImmunoResearch Laboratories, West Grove, PA, USA). The ligands used were: losartan (Abcam); angiotensin II, istradefylline (KW-6002), reserpine and ionomycin from Sigma-Aldrich (St. Louis, MO, USA).

### Plasmid constructs

To perform co-localization and BRET experiments, the A_2A_R constructs containing a cyan fluorescent protein (CFP; A_2A_R^CFP^), or the *Renilla* luciferase (*R*luc; A_2A_R^Rluc^) were used. The AT_1_R and GABA_B2_ receptor constructs containing a yellow fluorescent protein (YFP; AT_1_R^YFP^, GABA_B2_R^YFP^) were cloned, as previously described^63^.

### Animals

CD-1 mice (Charles River Laboratories and from the central animal facility of Federal University of Santa Catarina) and A_2A_R-KO mice developed in a CD-1 genetic background^64^ (animal facility of University of Barcelona) weighing 20–25 g were used. The University of Barcelona and Federal University of Santa Catarina Committee on Animal Use and Care approved the protocol. Animals were housed and tested in compliance with the guidelines described in the Guide for the Care and Use of Laboratory Animals^65^ and following the European Union directives (2010/63/EU). All efforts were made to minimize animal suffering and the number of animals used. All animals were housed in groups of five in standard cages with ad-libitum access to food and water and maintained under 12 h dark/light cycle (starting at 7:30 AM), 22 °C temperature, and 66% humidity (standard conditions).

### Cell culture

Human embryonic kidney (HEK)-293T cells were grown in Dulbecco’s modified Eagle’s medium (DMEM) (Sigma-Aldrich) supplemented with 1 mM sodium pyruvate, 2 mM L-glutamine, 100 U/mL streptomycin, 100 mg/mL penicillin and 5% (v/v) fetal bovine serum at 37 °C and in an atmosphere of 5% CO_2_. HEK-293T cells growing in 25 cm^2^ flasks or six-well plates containing 18 mm coverslips were used for western blot and fluorescence imaging, respectively. They were transiently transfected with the cDNA encoding the specified proteins using Polyethylenimine (Polysciences, Inc. Warrington, PA, USA).

### Fixed brain tissue preparation

Mice were anesthetized and perfused intracardially with 100–200 ml ice-cold 4% paraformaldehyde (PFA) in phosphate-buffered saline (PBS; 8.07 mM Na_2_HPO_4_, 1.47 mM KH_2_PO_4_, 137 mM NaCl, 0.27 mM KCl, pH 7.2). Brains were post-fixed in the same solution of PFA at 4 °C during 12 h. Coronal sections (25 μm) were processed using a vibratome (Leica Lasertechnik GmbH, Heidelberg, Germany). Slices were collected in Walter’s Antifreezing solution (30% glycerol, 30% ethylene glycol in PBS, pH 7.2) and kept at −20 °C until processing.

### Bioluminescence resonance energy transfer measurements

Bioluminescence resonance energy transfer (BRET) experiments in HEK-293T cells were performed as previously described^66^. In brief, HEK-293T expressing the indicated constructs were rapidly washed, detached, and resuspended in HBSS buffer (137 mM NaCl, 5 mM KCl, 0.34 mM Na_2_HPO_4_, 0.44 mM KH_2_PO_4_, 1.26 mM CaCl_2_, 0.4 mM MgSO_4_, 0.5 mM MgCl_2_, 10 mM HEPES, pH 7.4) containing 10 mM glucose. Cell suspensions (20 μg of protein) were distributed in 96-well microplate plates, 5 μM h-coelenterazine (NanoLight Technology, Pinetop, AZ, USA) was added and BRET determined in a POLARstar Optima plate-reader (BMG Labtech, Durham, NC, USA) as previously described^66^.

### Intracellular calcium determination

The AT_1_R-mediated intracellular Ca^2+^ accumulation was assessed by Fluo4-NW Calcium Assay Kit (Invitrogen, Carlsbad, CA, USA). Thus, transiently transfected HEK-293T cells were lifted and plated in 96-well black plates with transparent bottoms. Cells were incubated with the Fluo4-NW following the instructions of the manufacturer and washed with HBSS. Fluorescence signals were measured at 530 nm during 60 s while injecting Angiotensin II (50 nM) and ionomycin (5 μM) at seconds 5 and 40 respectively, using a POLARstar Optima plate-reader (BMG Labtech). The specific Angiotensin II-induced Fluo4 signal (F) was expressed as percentage of the signal elicited by ionomycin (Fi) in each set of experimental conditions^67^.

### Immunohistochemistry

Previously collected slices were washed three times in PBS, permeabilized with 0.3% Triton X-100 in PBS for 2 hours and rinsed back three times more with wash solution (0.05% Triton X-100 in PBS). The slices were then incubated with blocking solution (10% NDS in wash solution; Jackson ImmunoResearch Laboratories, Inc., West Grove, PA, USA) for 2 h at R.T. and subsequently incubated with the primary antibodies overnight at 4 °C. After two rinses (10 min each) with 1% NDS in wash solution, sections were incubated for 2 h at R.T. with the appropriate secondary antibodies conjugated with Alexa dyes (Invitrogen, Carlsbad, CA, USA), then washed (10 min each) two times with 1% NDS in wash solution and two more times with PBS, and mounted on slides. Fluorescence striatal images were obtained using a Leica TCS 4D confocal scanning laser microscope (Leica Lasertechnik GmbH).

### Proximity ligation *in situ* assay

Duolink *in situ* PLA detection Kit (Olink Bioscience, Uppsala, Sweden) was performed in a similar manner as immunohistochemistry explained above until the secondary antibody incubation step. The following steps were performed following the manufacturer’s protocol, as previously described^24, 40^. Fluorescence images were acquired on a Leica TCS 4D confocal scanning laser microscope (Leica Lasertechnik GmbH) using a 60x N.A. =1.42 oil objective from the selected area. High-resolution images were acquired as a z-stack with a 0.2 μm z-interval with a total thick of 5 μm. Nonspecific nuclear signal was eliminated from PLA images by substracting DAPI labeling. Analyze particle function from Image J (NIH) was used to count particles larger than 0.3 μm^2^ for PLA signal and larger than 100 μm^2^ to discriminate neuronal from glia nuclei^68^. For each image a number of oligomer particles and neuron nuclei was obtained and ratio among them was calculated.

### Computational modeling

#### Ligand docking

Crystal structures of AT_1_R (PDB id: 4ZUD) and A_2A_R (PDB id: 4EIY) were converted into apo *wt* forms by removing co-crystallized ligands and non-native fusion proteins i.e. cytochrome b562, building missing intracellular and extracellular loop sections with MODELLER v9.14^69^, and energy minimizing in the AMBER14SB force-field^70^ with CHIMERA v1.10.2^71^. The AT_1_R antagonist losartan and A_2A_R antagonist istradefylline were downloaded from PubChem^72^, energy-minimized in AMBER14SB force-field with CHIMERA and docked into respective receptor structures with Autodock4.2^25^ using default parameters. Grid points were generated to cover total orthosteric pocket volumes. The final docking conformations of losartan and istradefylline represented top hits identified by best predicted affinity (nM). These were checked to be consistent with previously reported binding modes of relevant co-crystallized antagonists^26, 28^. In particular, losartan was docked to interact with Arg167 and istradefylline was docked to interact with Asn253. Subsequent energy minimization of docked structures was performed with CHIMERA in the AMBER-14SB force-field.

#### Protein-protein docking

For generating homodimers of respective receptors: AT_1_R and A_2A_R with bound antagonists, two molecular dynamics (MD)-generated receptor-ligand monomers (see MD methods) of either AT_1_R or A_2A_R, in each case, were superimposed onto the A_2A_R homodimer crystal structure (PDB id: 4EIY), yielding an initial homodimer model, which was then submitted to the ROSIE webserver^37^ for protein-protein docking. For both AT_1_R and A_2A_R, the best docked homodimer was identified by three factors: best possible ROSETTA interface score (I_sc), lowest possible RMSD in relation to initial model, and acceptable membrane-compatible orientation. For construction of an AT_1_R-A_2A_R heterotetramer, two initial tetrameric arrangements were manually generated by combining respective MD-generated AT_1_R and A_2A_R homodimers (see MD section) in alternative ways: (i) where homodimers are arranged side-to-side in a rectangular-like configuration, where each homodimer subunit interacts with a subunit of the other homodimer (by respective TM1/2–5/6 helices), (ii) where homodimers are partially displaced with respect to one another creating a parallelogram-like configuration, where both subunits of one homodimer interact with a single subunit of the other homodimer (by respective TM4/5 helices). Both these alternative configurations were submitted to the ROSIE webserver for identification of the best possible tetrameric arrangement according to the same criteria implemented previously. For all protein-protein docking runs executed on the ROSIE webserver, default local parameters were used, i.e. perturbation of 3 Å between proteins, 8° of tilt, and 360° rotation around protein centers, with generation of 1000 docking solutions per case.

#### Molecular dynamics system setup

Five different systems were generated using the CHARMM-GUI web-based interface^73^, each in a POPC membrane and solvated with TIP3P water molecules: AT_1_R monomer with bound losartan, A_2A_R monomer with bound istradefylline, AT_1_R homodimer with bound losartan, A_2A_R homodimer with bound istradefylline, and AT_1_R-A_2A_R heterotetramer with bound antagonists. All receptor structures were orientated according to the OPM database^74^ entry: 4eiy. Charge neutralizing ions (0.15 M KCl) were introduced to each system. Parameters of membrane, water and protein were automatically generated by CHARMM-GUI^73^ according to CHARMM36 force-field^75^ with ligand parameters automatically generated according to CHARMM36 General Force Field^76–78^.

#### Molecular dynamics simulations

Molecular dynamics (MD) simulations of AT_1_R and A_2A_R were performed using the CHARMM36 force-field^75^ with ACEMD^79^ on specialized GPU-computer hardware, totaling 5 μs across systems. In detail, monomer AT_1_R/A_2A_R systems were equilibrated for 20 ns at 300 K and 1 atm, while AT_1_R/A_2A_R homodimers and heterotetramer systems were equilibrated for 50 ns under same conditions. During equilibration, positional harmonic restraints on protein and antagonist heavy atoms were progressively released over the first 8 ns and then continued without constraints. After equilibration, AT_1_R and A_2A_R monomers were subjected to unbiased production runs of 250 ns and 500 ns under same conditions, respectively. Likewise, AT_1_R and A_2A_R homodimers were subjected to unbiased production runs of 750 ns and 1.5 μs, respectively. The AT_1_R/A_2A_R heterotetramer was subjected to an unbiased production run of 2 μs. Simulation trajectories were analyzed using VMD software v1.9.2^80^.

### Reserpine-induced vacuous chewing movements

The VCM model of TD^48^ was induced in mice through two subcutaneous (s.c.) reserpine injections (1 mg/kg) administered with an interval of 48 h. Twenty-four hours after the last reserpine administration, mice were treated by intraperitoneal (i.p.) route with losartan (0.05–50 mg/kg) and/or istradefylline (0.03–0.06 mg/kg). VCM parameters were evaluated as previously described^81^ but with some modifications. Thus, the evaluation of VCM frequency consists of a manual counting of continuous single mouth openings in a vertical plane, not directed to a physical material. Mirrors were placed on the table and behind the glass cylinder (Ø 19 cm and 22 cm height) to allow observation of the orofacial movements when mice were not facing the observer. The evaluation of this parameter during 10 min was performed by a blind observer, 30 min after the pharmacological treatments administered 24 h after the second reserpine injection^81^.

### Statistics

The number of samples (n) in each set of experimental conditions is indicated in figure legends. Statistical analysis was performed by one-way ANOVA followed by Newman-Keuls *post-hoc* test or Student’s *t*-test when appropriate. Statistical significance was considered at *P* < 0.05.

## Electronic supplementary material

Supplementary data

## References

[1] Brunner HR, Chang P, Wallach R, Sealey JE, Laragh JH (1972). Angiotensin II vascular receptors: their avidity in relationship to sodium balance, the autonomic nervous system, and hypertension. J. Clin. Invest..

[2] Goa KL, Wagstaff AJ (1996). Losartan potassium: a review of its pharmacology, clinical efficacy and tolerability in the management of hypertension. Drugs.

[3] Hermann K, McDonald W, Unger T, Lang RE, Ganten D (1984). Angiotensin biosynthesis and concentrations in brain of normotensive and hypertensive rats. J. Physiol. (Paris)..

[4] Garrido-Gil P, Valenzuela R, Villar-Cheda B, Lanciego JL, Labandeira-Garcia JL (2013). Expression of angiotensinogen and receptors for angiotensin and prorenin in the monkey and human substantia nigra: an intracellular renin-angiotensin system in the nigra. Brain Struct. Funct..

[5] Saavedra JM (2005). Brain Angiotensin II: New Developments, Unanswered Questions and Therapeutic Opportunities. Cell. Mol. Neurobiol..

[6] Saavedra JM (2012). Angiotensin II AT(1) receptor blockers ameliorate inflammatory stress: a beneficial effect for the treatment of brain disorders. Cell. Mol. Neurobiol..

[7] Labandeira-GarcÃa JL (2014). Brain renin-angiotensin system and dopaminergic cell vulnerability. Front. Neuroanat..

[8] Grammatopoulos TN (2007). Angiotensin type 1 receptor antagonist losartan, reduces MPTP-induced degeneration of dopaminergic neurons in substantia nigra. Mol. Neurodegener..

[9] Zawada WM (2011). Generation of reactive oxygen species in 1-methyl-4-phenylpyridinium (MPP+) treated dopaminergic neurons occurs as an NADPH oxidase-dependent two-wave cascade. J. Neuroinflammation.

[10] Sonsalla PK (2013). The angiotensin converting enzyme inhibitor captopril protects nigrostriatal dopamine neurons in animal models of parkinsonism. Exp. Neurol..

[11] Agnati LF, Fuxe K, Zini I, Lenzi P, Hokfelt T (1980). Aspects on receptor regulation and isoreceptor identification. Med. Biol..

[12] Fuxe K (1983). Evidence for the existence of receptor–receptor interactions in the central nervous system. Studies on the regulation of monoamine receptors by neuropeptides. J. neural Transm..

[13] Gomes I (2016). G Protein–Coupled Receptor Heteromers. Annu. Rev. Pharmacol. Toxicol..

[14] Ciruela F (2004). Combining Mass Spectrometry and Pull-Down Techniques for the Study of Receptor Heteromerization. Direct Epitope–Epitope Electrostatic Interactions between Adenosine A 2A and Dopamine D 2 Receptors. Anal. Chem..

[15] Ferre S (2008). An Update on Adenosine A2A-Dopamine D2 receptor interactions. Implications for the Function of G Protein-Coupled Receptors. Curr. Pharm. Des..

[16] Müller T (2015). The safety of istradefylline for the treatment of Parkinson’s disease. Expert Opin. Drug Saf..

[17] Martínez-Pinilla E (2015). Dopamine D2 and angiotensin II type 1 receptors form functional heteromers in rat striatum. Biochem. Pharmacol..

[18] Pechlivanova DM, Georgiev VP (2002). Interaction of angiotensin II and adenosine A1 and A2A receptor ligands on the writhing test in mice. Pharmacol. Biochem. Behav..

[19] Tchekalarova J, Kambourova T, Georgiev V (2000). Long-term theophylline treatment changes the effects of angiotensin II and adenosinergic agents on the seizure threshold. Brain Res. Bull..

[20] Thakur S, Du J, Hourani S, Ledent C, Li JM (2010). Inactivation of adenosine A2A receptor attenuates basal and angiotensin II-induced ROS production by Nox2 in endothelial cells. J. Biol. Chem..

[21] Gomes CP (2008). Crosstalk between the signaling pathways triggered by angiotensin II and adenosine in the renal proximal tubules: Implications for modulation of Na^+^ -ATPase activity. Peptides.

[22] Lai EY, Patzak A (2009). Persson, a. E. G. & Carlström, M. Angiotensin II enhances the afferent arteriolar response to adenosine through increases in cytosolic calcium. Acta Physiol..

[23] Rongen Ga, Brooks SC, Ando SI, Abramson BL, Floras JS (1998). Angiotensin AT1 receptor blockade abolishes the reflex sympatho- excitatory response to adenosine. J. Clin. Invest..

[24] Fernández-Dueñas V (2015). Untangling dopamine-adenosine receptor-receptor assembly in experimental parkinsonism in rats. Dis. Model. Mech.

[25] Morris GM (2009). AutoDock4 and AutoDockTools4: Automated docking with selective receptor flexibility. J. Comput. Chem..

[26] Zhang H (2015). Structural Basis for Ligand Recognition and Functional Selectivity at Angiotensin Receptor. J. Biol. Chem..

[27] Ballesteros JA, Weinstein H (1995). Integrated methods for the construction of three-dimensional models of structure–function relations in G protein-coupled receptors. Methods Neurosci.

[28] Doré ASS (2011). Structure of the Adenosine A2A Receptor in Complex with ZM241385 and the Xanthines XAC and Caffeine. Structure.

[29] Thévenin D, Lazarova T, Roberts MF, Robinson CR (2005). Oligomerization of the fifth transmembrane domain from the adenosine A 2A receptor. Protein Sci..

[30] Canals M (2004). Homodimerization of adenosine A2A receptors: qualitative and quantitative assessment by fluorescence and bioluminescence energy transfer. J. Neurochem.

[31] Karip E, Turu G, Supeki K, Szidonya L, Hunyady L (2007). Cross-inhibition of angiotensin AT1 receptors supports the concept of receptor oligomerization. Neurochem. Int..

[32] AbdAlla S, Lother H, Langer A, el Faramawy Y, Quitterer U (2004). Factor XIIIA Transglutaminase Crosslinks AT1 Receptor Dimers of Monocytes at the Onset of Atherosclerosis. Cell.

[33] Hansen JL, Theilade J, Haunso S, Sheikh SP (2004). Oligomerization of Wild Type and Nonfunctional Mutant Angiotensin II Type I Receptors Inhibits G q Protein Signaling but Not ERK Activation. J. Biol. Chem..

[34] Fanelli F, Felline A (2011). Dimerization and ligand binding affect the structure network of A2A adenosine receptor. Biochim. Biophys. Acta - Biomembr..

[35] Gracia E (2011). A2A adenosine receptor ligand binding and signalling is allosterically modulated by adenosine deaminase. Biochem. J..

[36] Liu W (2012). Structural Basis for Allosteric Regulation of GPCRs by Sodium Ions. Science (80-.)..

[37] Lyskov S (2013). Serverification of Molecular Modeling Applications: The Rosetta Online Server That Includes Everyone (ROSIE). PLoS One.

[38] Bonaventura J (2015). Allosteric interactions between agonists and antagonists within the adenosine A2A receptor-dopamine D2 receptor heterotetramer. Proc. Natl. Acad. Sci. USA.

[39] Navarro G (2016). Quaternary structure of a G-protein-coupled receptor heterotetramer in complex with Gi and Gs. BMC Biol..

[40] Taura, J., Fernández-Dueñas, V. & Ciruela, F. Visualizing G Protein-Coupled Receptor-Receptor Interactions in Brain Using Proximity Ligation *In Situ* Assay. *Curr. Protoc. Cell Biol*. **67**, 17.17.1–17.17.16 (2015). 10.1002/0471143030.cb1717s67 26061241

[41] Ciruela F (2011). Adenosine receptor containing oligomers: their role in the control of dopamine and glutamate neurotransmission in the brain. Biochim. Biophys. Acta.

[42] Ferre S (2004). Adenosine A2A-dopamine D2 receptor-receptor heteromers. Targets for neuro-psychiatric disorders. Parkinsonism Relat. Disord..

[43] Ferre, S. *et al*. Adenosine A2A receptors and A2A receptor heteromers as key players in striatal function. *Front. Neuroanat*. **5** (2011). 10.3389/fnana.2011.00036 PMC3118889 21731559

[44] Parsons B, Togasaki DM, Kassir S, Przedborski S (1995). Neuroleptics up-regulate adenosine A2a receptors in rat striatum: implications for the mechanism and the treatment of tardive dyskinesia. J. Neurochem..

[45] Bishnoi M, Chopra K, Kulkarni SK (2006). Involvement of adenosinergic receptor system in an animal model of tardive dyskinesia and associated behavioural, biochemical and neurochemical changes. Eur. J. Pharmacol..

[46] Ivanova SA (2012). No involvement of the adenosine A2A receptor in tardive dyskinesia in Russian psychiatric inpatients from Siberia. Hum. Psychopharmacol. Clin. Exp..

[47] Andreassen OA, Jørgensen HA (2000). Neurotoxicity associated with neuroleptic-induced oral dyskinesias in rats. Implications for tardive dyskinesia?. Prog. Neurobiol..

[48] Turrone P, Remington G, Nobrega JN (2002). The vacuous chewing movement (VCM) model of tardive dyskinesia revisited: is there a relationship to dopamine D(2) receptor occupancy?. Neurosci. Biobehav. Rev..

[49] Fuxe K (2014). Moonlighting proteins and protein-protein interactions as neurotherapeutic targets in the G protein-coupled receptor field. Neuropsychopharmacology.

[50] Fuxe K, Marcellino D, Genedani S, Agnati L (2007). Adenosine A(2A) receptors, dopamine D(2) receptors and their interactions in Parkinson’s disease. Mov. Disord..

[51] Mendelsohn FA, Jenkins TA, Berkovic SF (1993). Effects of angiotensin II on dopamine and serotonin turnover in the striatum of conscious rats. Brain Res..

[52] Brown DC, Steward LJ, Ge J, Barnes NM (1996). Ability of angiotensin II to modulate striatal dopamine release via the AT1 receptor *in vitro* and *in vivo*. Br. J. Pharmacol..

[53] Villar-Cheda B (2010). Nigral and striatal regulation of angiotensin receptor expression by dopamine and angiotensin in rodents: Implications for progression of Parkinson’s disease. Eur. J. Neurosci.

[54] Villar-Cheda B (2014). Aging-related dysregulation of dopamine and angiotensin receptor interaction. Neurobiol. Aging.

[55] Borroto-Escuela DO (2010). Characterization of the A2AR-D2R interface: focus on the role of the C-terminal tail and the transmembrane helices. Biochem. Biophys. Res. Commun..

[56] Moriyama K, Sitkovsky MV (2010). Adenosine A2A Receptor Is Involved in Cell Surface Expression of A2B Receptor. J. Biol. Chem..

[57] Ciruela F (2010). G protein-coupled receptor oligomerization for what?. J. Recept. Signal Transduct. Res..

[58] Ferré S (2010). Adenosine-cannabinoid receptor interactions. Implications for striatal function. Br. J. Pharmacol..

[59] Yager LM, Garcia AF, Wunsch AM, Ferguson SM (2015). The ins and outs of the striatum: Role in drug addiction. Neuroscience.

[60] Jenner P (2005). Istradefylline, a novel adenosine A2A receptor antagonist, for the treatment of Parkinson’s disease. Expert Opin. Investig. Drugs.

[61] Jenner P (2009). Adenosine, adenosine A 2A antagonists, and Parkinson’s disease. Parkinsonism Relat. Disord..

[62] Muñoz A, Garrido-gil P, Dominguez-meijide A, Labandeira-garcia JL (2014). Angiotensin type 1 receptor blockage reduces L -dopa-induced dyskinesia in the 6-OHDA model of Parkinson’ s disease. Involvement of vascular endothelial growth factor and interleukin-1 β. Exp. Neurol..

[63] García-Negredo G (2014). Coassembly and coupling of SK2 channels and mGlu5 receptors. J. Neurosci..

[64] Ledent C (1997). Aggressiveness, hypoalgesia and high blood pressure in mice lacking the adenosine A2a receptor. Nature.

[65] Clark JD, Gebhart GF, Gonder JC, Keeling ME, Kohn DF (1997). Special Report: The 1996 Guide for the Care and Use of Laboratory Animals. ILAR J..

[66] Ciruela F, Fernández-Dueñas V (2015). GPCR oligomerization analysis by means of BRET and dFRAP. Methods Mol. Biol..

[67] Garcia-Negredo G (2014). Coassembly and Coupling of SK2 Channels and mGlu5 Receptors. J. Neurosci..

[68] Matamales M (2009). Striatal medium-sized spiny neurons: identification by nuclear staining and study of neuronal subpopulations in BAC transgenic mice. PLoS One.

[69] Sali A (1995). Comparative protein modeling by satisfaction of spatial restraints. Mol. Med. Today.

[70] Case DA (2005). The Amber biomolecular simulation programs. J. Comput. Chem..

[71] Pettersen EF (2004). UCSF Chimera–a visualization system for exploratory research and analysis. J. Comput. Chem..

[72] Kim S (2016). PubChem Substance and Compound databases. Nucleic Acids Res..

[73] Jo S, Kim T, Iyer VG, Im W (2008). CHARMM-GUI: a web-based graphical user interface for CHARMM. J. Comput. Chem..

[74] Lomize MA, Lomize AL, Pogozheva ID, Mosberg HI (2006). OPM: Orientations of Proteins in Membranes database. Bioinformatics.

[75] Huang J, MacKerell AD (2013). CHARMM36 all-atom additive protein force field: Validation based on comparison to NMR data. J. Comput. Chem..

[76] Vanommeslaeghe K (2010). CHARMM general force field: A force field for drug-like molecules compatible with the CHARMM all-atom additive biological force fields. J. Comput. Chem..

[77] Vanommeslaeghe K, Raman EP, MacKerell AD (2012). Automation of the CHARMM General Force Field (CGenFF) II: Assignment of Bonded Parameters and Partial Atomic Charges. J. Chem. Inf. Model..

[78] Vanommeslaeghe K, MacKerell AD (2012). Automation of the CHARMM General Force Field (CGenFF) I: Bond Perception and Atom Typing. J. Chem. Inf. Model..

[79] Harvey MJ, Giupponi G, Fabritiis G (2009). De. ACEMD: Accelerating Biomolecular Dynamics in the Microsecond Time Scale. J. Chem. Theory Comput..

[80] Humphrey, W., Dalke, A. & Schulten, K. VMD: visual molecular dynamics. *J. Mol. Graph*. **14**, 33–8, 27–8 (1996). 10.1016/0263-7855(96)00018-5 8744570

[81] Cunha AS (2016). Agmatine attenuates reserpine-induced oral dyskinesia in mice: Role of oxidative stress, nitric oxide and glutamate NMDA receptors. Behav. Brain Res..

