# Supplementary material for: Angiotensin II type 1/adenosine A2A receptor oligomers: a novel target for tardive dyskinesia
Source: Sci Rep. 2017 May 12;7:1857. doi: 10.1038/s41598-017-02037-z (PMC5431979; doi:10.1038/s41598-017-02037-z)
Supplement: Supplementary file 1 — Supplementary data [file 41598_2017_2037_MOESM1_ESM.doc]

**Supplementary Information**

**Angiontensin II type 1/adenosine A_2A_ receptor oligomer as target for tardive dyskinesia**

Paulo A. de Oliveira^1^,*, James A.R. Dalton^2^,*, Marc López-Cano^3,4^, Adrià Ricarte^2^, Xavier Morató^3,4^, Filipe C. Matheus^1^, Andréia S. Cunha^5^, Christa E. Müller^5^, Reinaldo N. Takahashi^1^, Víctor Fernández-Dueñas^3,4^, Jesús Giraldo^2,¥^, Rui D. Prediger^1,6,¥^, Francisco Ciruela^3,4,¥^

^1^Departamento de Farmacologia, Universidade Federal de Santa Catarina, Trindade, 88049-900, Florianópolis, SC, Brazil.

^2^Institut de Neurociències and Unitat de Bioestadística, Universitat Autònoma de Barcelona, Bellaterra, Spain; Network Biomedical Research Center on Mental Health (CIBERSAM).

^3^Unitat de Farmacologia, Departament de Patologia i Terapèutica Experimental, Facultat de Medicina, IDIBELL-Universitat de Barcelona, L’Hospitalet de Llobregat, Spain.

^4^Institut de Neurociències, Universitat de Barcelona, Barcelona, Spain.

^5^ PharmaCenter Bonn, Pharmaceutical Institute, Pharmaceutical Chemistry I, University of Bonn, Bonn, Germany.

^6^Programa de Pós-graduação em Neurociências, Centro de Ciências Biológicas, Universidade Federal de Santa Catarina, Trindade, 88049-900, Florianópolis, SC, Brazil.

*These authors contributed equally

**Running title:** AT_1_R/A_2A_R oligomers

^¥^**Corresponding authors:**

Francisco Ciruela (e-mail: [fciruela@ub.edu](mailto:fciruela@ub.edu))

Rui Prediger (e-mail: [rui.prediger@ufsc.br](mailto:ruidsp@hotmail.com))

Jesús Giraldo (e-mail: [Jesus.Giraldo@uab.es](mailto:Jesus.Giraldo@uab.es))

**Supplementary Figure 1**

**
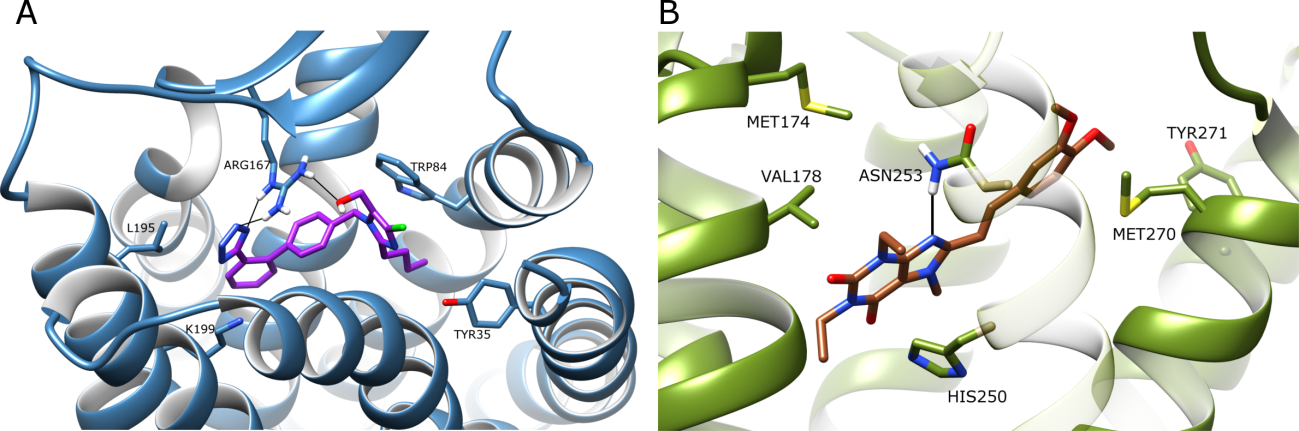
**

**Fig. S1.** Binding poses after docking and MD simulations of losartan (purple) in AT_1_R (PDB id: 4ZUD, blue ribbon) **(a)** and istradefylline (KW6002, brown) in A_2A_R (PDB id: 4EIY, green ribbon) **(b)**. Selected residues displayed and protein-ligand H-bonds represented by black lines.

**Supplementary Figure 2.**


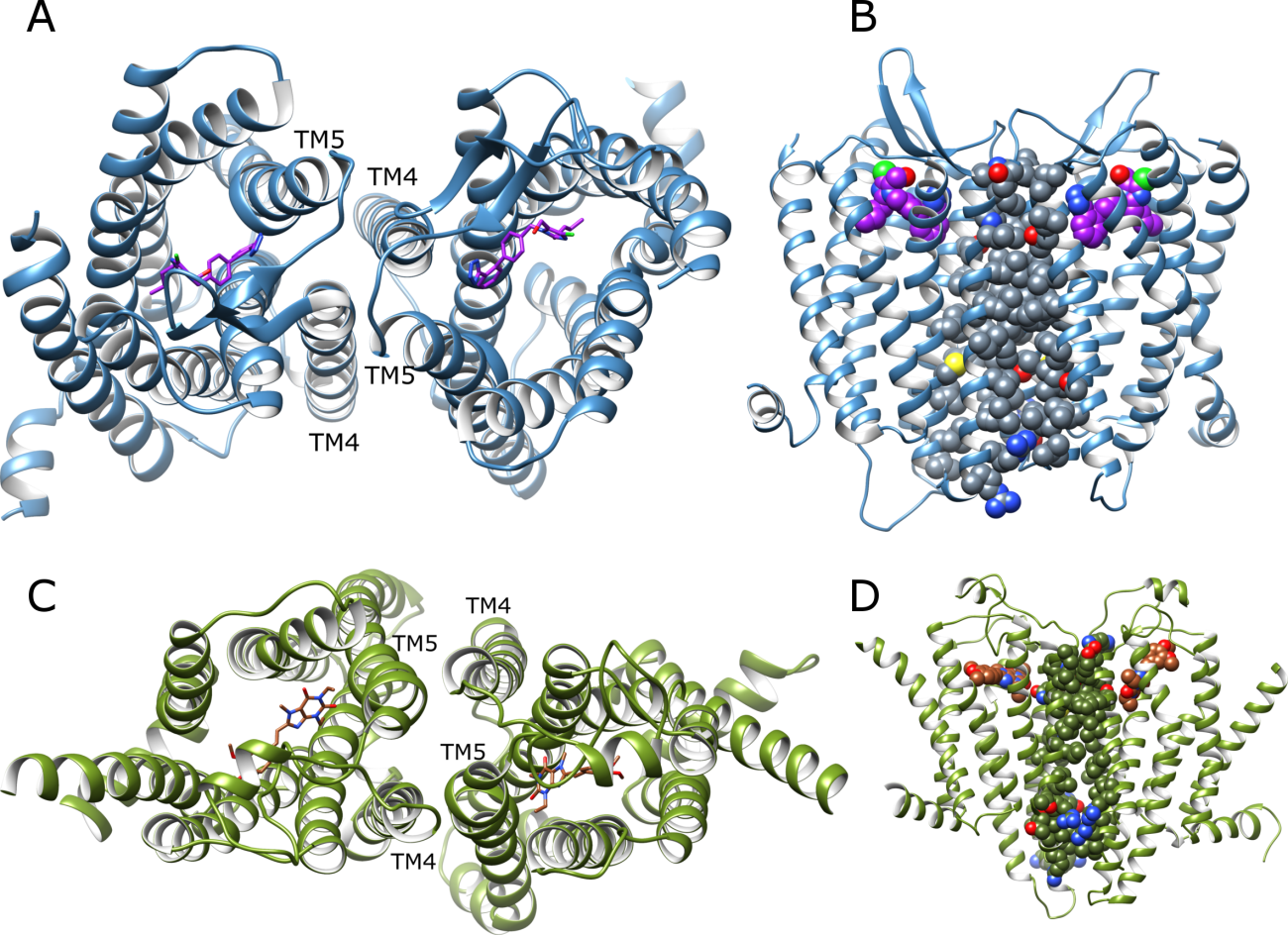


**Fig. S2.** After protein-protein docking and MD simulations: **(a-b)** homodimer of AT_1_R (blue ribbon) with bound losartan (purple), top and side view, **(c-d)** homodimer of A_2A_R (green ribbon) with bound istradefylline (brown), top and side view. In **(b)** and **(d)**, selected residues are displayed to show contact interfaces.

**Supplementary Figure 3.**


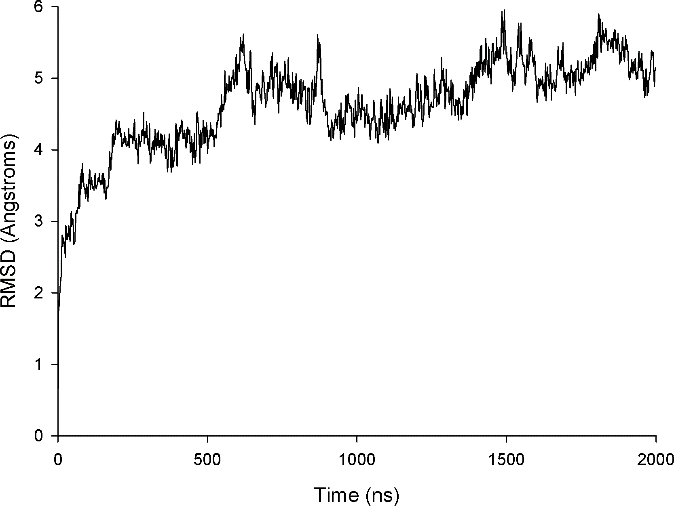


**Fig. S3.** RMSD of AT_1_R/A_2A_R heterotetramer (Cα atoms) over MD simulation.
